# Evaluation of *KRAS*, *NRAS* and *BRAF* mutations detection in plasma using an automated system for patients with metastatic colorectal cancer

**DOI:** 10.1371/journal.pone.0227294

**Published:** 2020-01-15

**Authors:** Claire Franczak, Andréa Witz, Karen Geoffroy, Jessica Demange, Marie Rouyer, Marie Husson, Vincent Massard, Céline Gavoille, Aurélien Lambert, Pauline Gilson, Nicolas Gambier, Julien Scala-Bertola, Jean-Louis Merlin, Alexandre Harlé

**Affiliations:** 1 Institut de Cancérologie de Lorraine, Service de Biopathologie, Vandoeuvre les Nancy, France; 2 Institut de Cancérologie de Lorraine, Département d’oncologie médicale, Vandoeuvre les Nancy, France; 3 Université de Lorraine, CNRS UMR 7039 CRAN, Institut de Cancérologie de Lorraine, Service de Biopathologie, Nancy, France; 4 CHRU Nancy, Department of Clinical Pharmacology and Toxicology, Nancy, France; 5 Université de Lorraine, UMR 7365 CNRS-UL, IMoPA, Vandœuvre-lès-Nancy, France; Sapporo Ika Daigaku, JAPAN

## Abstract

**Background:**

Cell-free DNA detection is becoming a surrogate assay for tumor genotyping. Biological fluids often content a very low amount of cell-free tumor DNA and assays able to detect very low allele frequency mutant with a few quantities of DNA are required. We evaluated the ability of the fully-automated molecular diagnostics platform Idylla for the detection of *KRAS*, *NRAS* and *BRAF* hotspot mutations in plasma from patients with metastatic colorectal cancer (mCRC).

**Materials and methods:**

First, we evaluated the limit of detection of the system using two set of laboratory made samples that mimic mCRC patient plasma, then plasma samples from patients with mCRC were assessed using Idylla system and BEAMing digital PCR technology.

**Results:**

Limits of detection of 0.1%, 0.4% and 0.01% for *KRAS*, *NRAS* and *BRAF* respectively have been reached. With our laboratory made samples, sensitivity up to 0.008% has been reached. Among 15 patients’ samples tested for *KRAS* mutation, 2 discrepant results were found between Idylla and BEAMing dPCR. A 100% concordance between the two assays has been found for the detection of *NRAS* and *BRAF* mutations in plasma samples.

**Conclusions:**

The Idylla system does not reach as high sensitivity as assays like ddPCR but has an equivalent sensitivity to modified NGS technics with a lower cost and a lower time to results. These data allowed to consider the Idylla system in a routine laboratory workflow for *KRAS*, *NRAS* and *BRAF* mutations detection in plasma.

## Introduction

Presence of cell-free nucleic acids (cfNA) in plasma has been described in 1948 by Mandel and Métais [1]. In 1977, Leon *et al*. described that concentration of cell free DNA (cfDNA) is higher in plasma of patients with cancer [2]. Cell-free tumor DNA (ctDNA) is mostly shed in body fluids by apoptosis, necrosis and active mechanisms. ctDNA is also found in several types of micro vesicles like exosomes and micro particles secreted by most cells including tumor cells [3]. ctDNA is commonly more fragmented than cfDNA deriving from healthy cells (160–180 bp) and fragments have a size mainly smaller than 145 nucleotides [4,5].

The research of *KRAS* and *NRAS* (*RAS* genes) mutations is highly important since the existence of a mutation on codons 12, 13, 59, 61, 117 or 146 is known as a resistance marker to anti-EGFR monoclonal antibodies (*i*.*e*. cetuximab and panitumumab) associated with chemotherapy in the management of patients with metastatic colorectal cancer (mCRC) [6]. Moreover, the presence of a V600E *BRAF* mutation is recognized as a poor prognosis factor [7], thus assessment of *KRAS*, *NRAS* and *BRAF* has become a standard for the management of patients with mCRC.

Formalin-fixed paraffin embedded (FFPE) tissue is recognized as the gold standard for the research of *RAS* and *BRAF* mutations. Tumor biopsy is not always possible and is an invasive procedure for patients with cancer. The patient’s follow-up and the determination of minimal residual disease also require iterative biopsies, which is not possible nor ethical using tissue. Moreover, because of the formalin fixation process, DNA extracted from FFPE tissues is sometimes too fragmented or of bad quality. The assessment of *RAS* and *BRAF* mutations using ctDNA extracted from plasma could be a fair alternative for patient quality of life improvement since a blood sample is an easier and less invasive procedure than a tissue biopsy.

ctDNA detected in plasma has been described as representative of tumor heterogeneity and several studies showed a good concordance with tissue samples. In the study conducted by Thierry *et al*., samples from 140 patients with mCRC have been analyzed and a concordance of 72% was showed between *KRAS* exon 2 status found in plasma and FFPE tissue [8,9]. In the RASANC prospective study, *RAS* status was determined using next-generation sequencing (NGS) on 412 paired plasma and tumor samples. An excellent concordance (kappa coefficient 0.71 [95% CI: 0.64–0.77] and accuracy 85.2% [95% CI: 81.4–88.5]) were found between plasma and tissue [10]. These different studies allowed considering the use of liquid biopsy but with a necessity of tumor tissue testing in case of negative results in plasma.

The Idylla platform is a CE-IVD fully-integrated system based on real-time polymerase chain reaction (PCR). This system has already been validated for the determination of *RAS* and *BRAF* mutations using FFPE tissues [11–15] and for the *BRAF* hotspot mutation detection in plasma samples [16–19].

ctDNA can represent between 0.01% and 90% of the cfDNA extracted from plasma, thus a very sensitive assay is needed for a reliable detection of low amount of ctDNA and/or low variant allele frequency [20]. In this study, we evaluated the ability and the limit of detection (LOD) of the Idylla system for the detection of *KRAS*, *NRAS* and *BRAF* mutations in plasma using laboratory-made samples (DNA from cell-line and from commercial controls) that mimic patients and samples from patients with mCRC.

## Materials and methods

### DNA from characterized cell lines

Details of ATCC cell lines used to obtain DNA with characterized mutations. Cell line details are described in Table 1 and culture conditions in S1 Table.

**Table 1:** Details of cell-lines used to create our plasma samples.

Cell-line	ATCC® reference	Gene	Exon	Reference Sequence	Coding DNA Sequence mutation Amino Acid mutation	Mutation
ML-2	CVCL_1418T^M^	*KRAS*	4	NM_033360.2	c.436G>A p.(Ala146Thr)	Heterozygous
SW620	CCL-227^TM^	*KRAS*	2	NM_033360.2	c.35G>T p.(Gly12Val)	Homozygous
HT29	HTB-38^TM^	*BRAF*	15	NM_004333.4	c.1799T>A p.(Val600Glu)	Heterozygous
MZ2	CVCL-1435^TM^	*NRAS*	3	NM_002525.4	c.181C>A p.(Gln61Lys)	Heterozygous

DNA extracted from characterized cell lines (Table 1) was measured using the Qubit^®^ dsDNA HS assay kit (which allows detection of concentration between 0.01 and 100 ng/μL) and the Qubit^®^ 3.0 Fluorometer (ThermoFisher Scientific Inc, Massachusetts, USA). Extracted DNA was then diluted in TE buffer at 4ng/μL. Concentrations of the diluted samples were finally assessed using Qubit^®^ prior to fragmentation.

**Fig 1:**
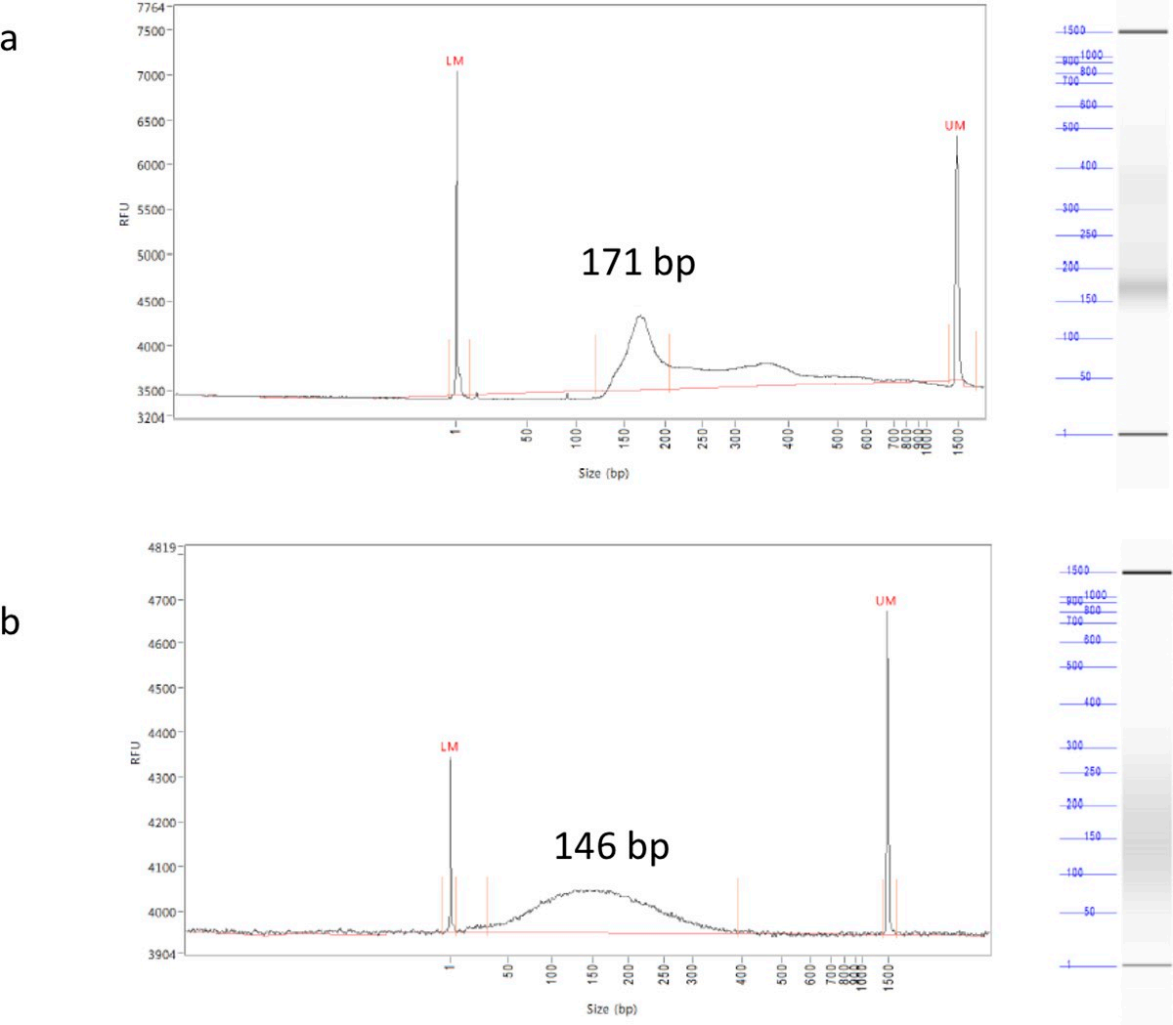
DNA size profiles obtained using fragment analyzer^®^. **a**. DNA fragments profile of DNA extracted from plasma of patient with metastatic colorectal cancer **b.** DNA fragments profile of DNA extracted from ML-2 cell line after fragmentation with Covaris®.

DNA was then fragmented using Covaris^®^ M220 Focused-Ultrasonicator (Covaris, Massachusetts, USA) duration 150 seconds, peak incident power 75.0 Watt, duty factor 26.0% and burst 150. Fragments sizes were then determined using Fragment analyzer^®^ (Advanced Analytical, Ankeny, USA) and DNF-477 High Sensitivity Small Fragment Analysis Kit (1 bp– 1 500 bp). According to Thierry *et al*, which report that ctDNA is more fragmented than cfDNA deriving from healthy cells, with a size mainly smaller than 145 bp, targeted DNA fragments size was 145 bp [3]. Fig 1 shows fragmentation profile for ML-2 cell line; similar data were obtained for the 3 other cell lines.

Commercial plasma (Dutscher S4180-500, Brumath, France) obtained from healthy donor was defrosted and centrifuged 10 minutes at 20°C– 6,000G.

This commercial plasma doesn’t contain ctDNA but naturally contains cfDNA whose concentration was determined using Qubit® prior addition of ctDNA solution (Table 2).

To obtain several plasma samples with different ctDNA concentration, different volumes of fragmented DNA (300 copies/ng) solution were spiked in 1 mL of commercial plasma. A concentration range was obtained for each cell-line (Table 2, S2 and S3 Tables).

**Table 2:** Limit of detection of one *KRAS* exon 2 and one *KRAS* exon 4 mutations by ctKRAS mutation assay and detection of one *BRAF* codon 600 mutation and one *NRAS* exon 3 mutation by ctNRAS-BRAF mutation assay on samples mimicking plasma.

Gene mutation	cfDNA concentration*	ctDNA concentration^†^	Mutated ctDNA concentration^‡^	cfDNA total concentration^§^	Ratio mutated copies / wild-type copies	Cq wild-type (control)^||^	Cq mutated ^||^	Mutation interpretation
	ng/mL	ng/mL	ng/mL	ng/mL	%			
	copies /mL	copies /mL	copies / mL	copies /mL				
***KRAS*** **p.(Ala146Thr)**	460.0	9.00 x 10^−2^	4.50 x 10^−2^	460.09	1/10 000	27.7	39.2	Detected
	138 000	28	14	138 028	0.010%			
	460.0	7.70 x 10^−2^	3.85 x 10^−2^	460.08	1/12 000	29.0	39.6	Detected
	138 000	23	11.5	138 23	**0.009%**			
	460.0	7.40 x 10^−2^	3.70 x 10^−2^	460.07	1/12 500	29.8	-	Not detected
	138 000	22	11	138 022	0.008%			
***KRAS*** **p.(Gly12Val)**	460.0	4.00 x 10^−2^	4.00 x 10^−2^	460.04	1/11 500	28.1	38.3	Detected
	138 000	12	12	138 012	0.009%			
	460.0	3.50 x 10^−2^	3.50 x 10^−2^	460.035	1/13 000	27.9	32.8	Detected
	138 000	10.5	10.5	138 010	**0.008%**			
***BRAF*** **p.Val600Glu**	460.0	14.00 x 10^−2^	7.00 x 10^−2^	460.014	1/6 500	37.8	53.4	Detected
	138 000	42	21	138 42	0.015%			
	460.0	10.50 x 10^−2^	5.25 x 10^−2^	460.0525	1/8 750	38.1	55.3	Detected
	138 000	32	16	138 032	**0.011%**			
	460.0	10.30 x 10^−2^	5.15 x 10^−2^	460.010	1/9 000	37.7	-	Not detected
	138 000	30	15	138 030	0.011%			
***NRAS*** **p.Gln61Lys**	460.0	14.00 x 10^−2^	7.00 x 10^−2^	460.014	1/6 500	37.8	55	Detected
	138 000	42	21	138 042	**0.015%**			
	460.0	13.00 x 10^−2^	6.50 x 10^−2^	460.013	1/7 000	37.7	-	Not detected
	138 000	40	20	138 040	0.014%			
	460.0	12.66 x 10^−2^	6.33 x 10^−2^	460.012	1/7 250	37.6	-	Not detected
	138 000	38	19	138 038	0.014%			

* cfDNA concentration: concentration of cfDNA in commercial plasma

^†^ ctDNA concentration: concentration of ctDNA added in commercial plasma

^‡^ mutated ctDNA concentration = ctDNA concentration for homozygous mutation and ctDNA concentration/2 for heterozygous mutation

^§^ cf DNA total concentration = cfDNA concentration + ctDNA concentration. cfDNA total number of copies = cfDNA number of copies + ctDNA number of copies

^||^ cycle quantification.

### ctDNA from commercial panel control

To complete the panel of tested mutations, we used AcroMetrix Idylla ctRAS verification Panel (ThermoFisher Scientific Inc). This commercial panel of controls is composed of 9 vials containing one mutation each. Each vial contains 167 ng of fragmented DNA (160 +/- 24 bp) in normal human EDTA plasma (total volume of 1.1 mL). DNA is 5% (+/-0.50%) mutated for p.(Gly12Asp), p.(Gly13Asp), p.(Gly12Ser), p.(Gly12Val) *KRAS* mutations and p.(Val600Glu) *BRAF* mutation and 10% (+/-1.5%) mutated for p.(Gly12Asp), p.(Gly12Val), p.(Gln61Arg) and p.(Gln61Lys) *NRAS* mutations.

To assess the limits of detection of the Idylla system, these controls were diluted in different volumes of commercial plasma (Dutscher) to obtain a concentrations range of mutated allele fractions (Table 3).

**Table 3:** Limit of detection of four *KRAS* exon 2 mutations by ctKRAS mutation assay and limit of detection of one *BRAF* codon 600 mutation, two *NRAS* exon 2 mutations and two *NRAS* exon 3 mutations by ctNRAS-BRAF mutation assay on a commercial controls.

	Volume of control (μL) in 1 mL of commercial plasma	Number of Mutated copies in the sample	Ratio mutated copies / wild-type copies %	Cq wild-type (control)	Cq mutated	Mutation interpretation
***KRAS*** **p.(Gly12Asp)**	60.60	138	0.1%	24.7	30.61	Detected
	30.30	69	**0.05%**	25.5	33.36	Detected
	12.12	28	0.02%	26.3	-	Not detected
***KRAS*** **p.(Gly12Ser)**	60.60	138	0.1%	25.3	32.6	Detected
	30.30	69	**0.05%**	26.0	34.1	Detected
	15.15	35	0.025%	26.5	-	Not detected
***KRAS*** **p.(Gly12Val)**	15.15	35	0.025%	26.4	36.5	Detected
	9.09	21	**0.015%**	27.0	36.97	Detected
	6.06	14	0.01%	27.2	-	Not detected
***KRAS*** **p.(Gly13Asp)**	90.90	197	0.15%	25.2	30.3	Detected
	60.60	138	**0.1%**	24.9	30.8	Detected
	45.45	104	0.075%	25.6	-	Not detected
***BRAF*** **p.(Val600Glu)**	6.06	14	0.01%	37.8	55.1	Detected
	4.54	10	**0.0075%**	37.1	52.7	Detected
	3.03	7	0.005%	37.3	-	Not detected
***NRAS*** **p.(Gly12Asp)**	151.50	690	0.5%	34.6	47.0	Detected
	121.20	552	**0.4%**	35.0	50.6	Detected
	90.90	414	0.3%	35.1	-	Not detected
***NRAS*** **p.(Gly12Val)**	90.90	414	0.3%	35.2	45.1	Detected
	30.30	138	**0.1%**	36.5	50.7	Detected
	15.15	69	0.05%	36.7	-	Not detected
***NRAS*** **p.(Gln61Arg)**	90.90	414	0.3%	35.3	44.3	Detected
	30.30	138	**0.1%**	37.6	46.4	Detected
	15.15	69	0.05%	37.4	-	Not detected
***NRAS*** **p.(Gln61Lys)**	121.20	552	0.4%	35.1		Detected
	90.90	414	**0.3%**	35.8	48.42	Detected
	75.75	345	0.25%	35.5	-	Not detected

### Patient samples

Patients’ samples were selected in January 2018 from the Institut de Cancérologie de Lorraine biobank (Nancy, France) (CircuLOR-1 study (NCT02751177)). Methods were performed in accordance with the relevant guidelines and regulations and approved by the ethical and scientific board of Institut de Cancérologie de Lorraine. This study has been specifically approved by the ethical and scientific board of Institut de Cancérologie de Lorraine. All samples were anonymized prior to analysis. Plasma samples were collected from patients with a mCRC with a requested *KRAS*, *NRAS* or *BRAF* genotyping. cfDNA was extracted from plasma using the QIAamp Circulating Nucleic Acid Kit (Qiagen, Hilden, Germany). Then, BEAMing method was performed with the OncoBEAM CRC1kit (Sysmex Inostics, Hamburg, Germany). This technique consists on a hybridization between DNA-coated beads and a sequence specific fluorescein-labeled probe which are then analyzed by flow cytométrie [21,22]. Data are processed by a software and results then reported as “no mutation detected” or “mutation detected”.

In our study, mutation detection with Idylla platform was performed on 15 samples with ctKRAS cartridge and in 3 samples with ctNRAS-BRAF cartridge.

Discrepant samples were assessed using a third assay. DNA was extracted from plasma using the QIAamp® Circulating Nucleic Acid kit (Qiagen, Hilden, Germany), libraries were then prepared with the custom STS 51-Gene kit (Sophia genetics, Saint-Sulpice, Switzerland), a capture-based target enrichment kit. Sequencing was performed on MiSeq (Illumina, San Diego, USA) and results were treated using Sophia DDM® software (Sophia genetics).

### Idylla platform

Idylla platform (Biocartis, Mechelen, Belgium) is a fully cartridge-based automated platform and uses microfluidics processing with all reagents on-board.

ctKRAS cartridge can detect 21 mutations on codons 12, 13, 59, 61, 117 et 146 of *KRAS* gene in plasma samples. ctNRAS-BRAF cartridge can detect 18 mutations in codons 12, 13, 59, 61, 117, 146 of *NRAS* gene, 5 mutations in codon 600 of the *BRAF* gene (S4 Table).

One milliliter of plasma and 20 μL of proteinase K were added in the cartridge. Proteinase K was added proteins hydrolyzation, including fibrin which could potentially interfere with DNA analysis. The cartridge was then sealed and inserted in the instrument. Inside the cartridge, nucleic acids are amplified using real-time PCR coupled with a fluorophore-based detection system. Idylla console autoanalyses the PCR curve to determine the presence or absence of a mutation and the results are presented as either “no mutation detected” or “mutation detected” [14].

Cycle quantification (Cq) of the control and of the mutated sample are available on the result sheet.

## Results

### Laboratory made samples that mimic plasma

A range of dilutions between 2.30 x 10^−2^ ng/mL (7 copies/mL) and 2.30 x 10^−1^ ng/mL (69 copies/mL) (13 points) and between 3.50 x 10^−2^ ng/mL (10.5 copies/mL) and 4.00 x 10^−2^ ng/mL (12 copies/mL) (3 points) were prepared for p.(Ala146Thr) and p.(Glu12Val) *KRAS* mutations respectively.). The range obtained was between 3.50 x 10^−2^ ng/mL (10.5 copies/mL) and 7.00 x 10^−2^ ng/mL (21 copies/mL) for p.(Val600Glu) *BRAF* mutation (7 points) and between 1.67 x 10^−2^ ng/mL (5 copies/mL) and 7.00 x 10^−2^ ng/mL (21 copies/mL) for p.(Gln61Lys) *NRAS* mutation (7 points). All concentrations are described in the S2 and S3 Tables.

Sensitivity were 10.5 copies/mL for p.(Glu12Val) *KRAS* mutation, 11.5 copies/mL for p.(Ala146Thr) *KRAS* mutation, 16 copies/mL for p.(Val600Glu) *BRAF* mutation and 21 copies/mL for p.(Gln61Lys) *NRAS* mutation

Considering wild type DNA present in our samples, sensitivity of ctKRAS mutation assay was 1 mutated copy/12 000 wild-type copies for *KRAS* mutation (Table 2). Sensitivity of ctNRAS-BRAF mutation assay was 1 mutated copy/8750 wild-type copies and 1 mutated copy/6500 wild-type copies for *BRAF* and *NRAS* mutations respectively (Table 2).

No mutation has been detected in negative controls (plasma from healthy donor used for samples preparation).

### Commercial panel control

Different volumes of control have been used to obtain allele frequencies mutant between 0.01% (14 copies /mL) and 0.1% (138 copies/mL) for *KRAS* p.(Gly12Asp) (4 points), 0.015% (21 copies/mL) and 0.15% (197 copies/mL) for *KRAS* p.(Gly13Asp) (3 points), 0.01% (14 copies/mL) and 0.1% (138 copies/mL) for *KRAS* p.(Gly12Val) (5 points) and 0.025% (14 copies/mL) and 0.1% (138 copies/mL) for *KRAS* p.(Gly12Ser) (6 points). Different volumes of *NRAS* mutated controls have been used to obtain allele frequencies mutant between 0.1% (138 copies/mL) and 0.5% (690 copies/mL) for *NRAS* p.(Gly12Asp) (5 points), 0.05% (69 copies/mL) and 0.5% (690 copies/mL) for *NRAS* p.(Gly12Val) mutations (4 points), 0.05% (69 copies/mL) and 0.3% (414 copies/mL) for *NRAS* p.(Gln61Arg) (3 points) and between 0.1% (138 copies/mL) and 0.4% (552 copies/mL) for *NRAS* p.(Gln61Lys) mutation (5 points). All concentrations are described in S5 Table. *BRAF* p.(Val600Glu) mutation has been tested between 0.005% (7 copies/mL) and 0.01% (14 copies/mL) (3 points). All concentrations are described in S6 Table.

LOD were 21 copies/mL for *KRAS* p.(Gly12Val), 69 copies/mL for *KRAS* p.(Gly12Asp) and *KRAS* p.(Gly12Ser) mutations and 138 copies for *KRAS* p.(Gly13Asp) mutations (Table 3).

LOD was 10 copies/mL for p.(Val600Glu) *BRAF* mutation.

LOD were 138 copies/mL for *NRAS* p.(Gly12Val) mutation, 414 copies/mL for *NRAS* p.(Gln61Lys) mutation and 552 copies/mL for *NRAS* p.(Gly12Asp) and p.(Gln61Arg) mutations (Table 3).

### Patient’s samples

*KRAS* mutations have been detected in 12/15 samples with ctKRAS cartridges and 14/15 samples with BEAMing dPCR (Tables 4 and 5). One sample was found wild-type for *KRAS*, *NRAS* and *BRAF* with both techniques. Idylla system and BEAMing dPCR both found 2 *NRAS* mutations (Tables 4 and 5). One *BRAF* mutation was detected by both Idylla system and BEAMing dPCR (Tables 4 and 5).

**Table 4:** Results obtained with BEAMing and Idylla assays with ΔCq data for mutated samples.

Patient	BEAMing	Idylla	ΔCq mutated Idylla
1	KRAS exon 2 codon 12	KRAS p.(Gly12Asp)	6,2
2	KRAS exon 2 codon 12	KRAS p.(Gly12Asp)	5,71
3	KRAS exon 2 codon 13	KRAS p.(Gly13Asp)	4,52
4	KRAS exon 2 codon 13	KRAS p.(Gly13Asp)	6,23
5	KRAS exon 3 codon 61	KRAS p.(Gln61His)	5,77
6	KRAS exon 3 codon 61	KRAS p.(Gln61Ar) ou p.(Gln61Leu)	4,74
7	KRAS exon 4 codon 146	No mutation detected	/
8	KRAS exon 4 codon 146	No mutation detected	/
9	KRAS exon 2 codon 12	KRAS p.(Gly12Asp)	5,21
10	KRAS exon 4 codon 146	KRAS p.(Ala146Pro) ou p.(Ala146Thr) ou p.(Ala146val)	8,84
11	KRAS exon 2 codon 12	KRAS p.(Gly12Asp)	8,95
12	KRAS exon 2 codon 12	KRAS p.(Gly12Val)	2,85
13	KRAS exon 2 codon 13	KRAS p.(Gly13Asp)	4,39
14	KRAS exon 2 codon 13	KRAS p.(Gly13Asp)	4,34
15	Wild-type for KRAS	No mutation detected	/
16	NRAS exon 3 codon 61	NRAS p.(Gln61Arg) or p.(Gln61Lys)	12,67
17	NRAS exon 2 codon 13	NRAS p.(Gly13Arg) or p.(Gly13Val)	11,65
18	BRAF codon 600	BRAF p.(Val600Glu)	12,19

**Table 5:** Concordance between BEAMing dPCR and Idylla system for *KRAS*, *NRAS* and *BRAF* mutation detection in plasma of mCRC patients.

***KRAS* (n = 15)**							
	**BEAMing dPCR**						
	p.(Gly12Asp)	p.(Gly12Val)	p.(Gly13Asp)	p.(Gln61His)	p.(Gln61Arg) or p.(Gln61Leu)	p.(Ala146Pro) or p.(Ala146Thr) or p.(Ala146Val)	WT
**ctKRAS Idylla cartridge**							
p.(Gly12Asp)	4						
p.(Gly12Val)		1					
p.(Gly13Asp)			4				
p.(Gln61His)				1			
p.(Gln61Arg) or p.(Gln61Leu)					1		
p.(Ala146Pro) or p.(Ala146Thr) or p.(Ala146Val)						1	
WT						2	1
Total	4	1	4	1	1	3	1
***NRAS* (n = 2)**							
	**BEAMing dPCR**						
	p.(Gln61Arg) or p.(Gln61Lys)	p.(Gly13Arg) or p.(Gly13Val)	WT				
**ctNRAS-BRAF Idylla cartridge**							
p.(Gln61Arg) or p.(Gln61Lys)	1	0					
p.(Gly13Arg) or p.(Gly13Val)	0	1					
WT	0	0	0				
Total	1	1	0				
***BRAF* (n = 1)**							
	**BEAMing dPCR**						
	p.(Val600Glu) or p.(Val600Asp)	WT					
**ctNRAS-BRAF Idylla cartridge**							
p.(Val600Glu) or p.(Val600Asp)	1	0					
WT	0	0					
Total	1	0					

Two discrepant samples were found. Both samples were found carrying a codon 146 mutation of *KRAS* gene detected with BEAMing and not detected with Idylla. Among these 2 samples, only one was found to carry a codon 146 mutation of *KRAS* and the other one was found to carry no mutation using NGS.

## Discussion

In this study, two different set of mutated DNA (from cell lines and from commercial controls) have been used to obtain samples that mimic blood samples from patients with mCRC. We have obtained samples with different mutant allele frequencies for 5 different *KRAS* mutations, 1 *BRAF* mutation and 4 different *NRAS* mutations. Then the use of Idylla system has been evaluated on 18 plasma samples from patients with mCRC. According to the manufacturer technical sheet the LODs of the Idylla system depend on the mutation and are between 0.2% and 2.8% for *KRAS* mutations, between 0.2% and 0.5% for *BRAF* mutations and between 0.6% and 2.5% for *NRAS* mutations (with a background of 50 000 WT copies). Our panel has been developed to cover several mutations with different LOD. We finally obtained LOD of 0.1%, 0.4% and 0.01% for *KRAS*, *NRAS* and *BRAF* respectively. To select mutations, we focused on exon 2 codons 12 and 13 mutations which represent the majority of *KRAS* mutations in mCRC [23,24]. To assess the ability of Idylla system to detect less prevalent hotspot mutations, we also added a *KRAS* exon 4 mutation p.(Ala146Thr). Because they also are the most described mutations in mCRC, *BRAF* codon 600 and *NRAS* exon 2 codon 12 and exon 3 codon 61 were also chosen for this evaluation [7,23]. It is important to notice that the Cq are very high for samples with very low VAF which was expectable. The manufacturer of the Idylla system warns that there is a risk of false negative result with their system for Cq higher than 25.5 for *KRAS* and 35.5 for *NRAS/BRAF*. Since the aim of this study was to estimate the LOD of the system, we made the choice to retain the samples with Cq higher than the maximum Cq recommended by the manufacturer. In clinical settings, it seems important to us that the samples have to be analyzed with another assay when Cq are higher than the maximum recommended Cq.

Samples used to mimic mCRC patient’s plasma samples have several limitations since they have been elaborated in ideal conditions. In our study, we spiked fragmented DNA in plasma samples, thus 100% of them were containing cfDNA which is not always the case in the “real-life” because of the intermittent shedding of DNA by the tumor. It has been shown that blood samples from patients with cancer may sometimes not contain ctDNA [25]. Tumor cells and ctDNA have also some cancer-associated molecular characteristics such as single-nucleotide mutation or a specific methylation profile that we were not able to reproduce *in vitro* [26,27]. For ctDNA use in clinical applications, adding the detection of these epigenomics or genomics features may ensure the presence of ctDNA in the sample.

We used DNA of good quality extracted from cell-lines and mechanical fragmentation to prepare 145bp DNA fragments. Quality of plasma and DNA concentrations were also well controlled. We note in this way discrepancies when using a commercial panel of controls for LOD determination. For example, *NRAS* p.(Gln61Lys) mutation was detected in our laboratory made samples with 21 mutated copies/mL of plasma whereas 414 copies/mL of commercial control DNA were necessary for mutation detection. Perhaps freezing / thawing cycles of commercial controls alter DNA quality. Moreover, we have to considered declared concentrations and pipetting uncertainty. These two set of samples highlight that mutation detection depends on several preanalytical factors. To understand these discrepancies it would have been necessary to measure DNA control fragment size or concentration. For a real-life application of ctDNA detection in patients with cancer, we have to consider that various pre-analytical factors can influence samples quality. Sample handling as centrifugation procedures or storage conditions can drastically influence samples quality. Thus, a standardized pre-analytical protocol is required [28]. cfDNA is mainly present on structures like nucleosomes which protect them from nuclease degradation [3]. To prevent contamination of ctDNA by DNA shed by white blood cells, several specific tubes as Streck BCT cfDNA blood collection tubes are available and allow samples handling up to 5 days [29,30].

To translate our laboratory study into clinical practice, we tested 18 plasma samples from patients with mCRC with various mutations identified using BEAMing dPCR. Among the 18 plasma samples tested, 2 discrepant results were found. One *KRAS* exon 4, codon 146 mutation was detected with BEAMing and not by Idylla system and NGS. At this point, it is not possible to know whether this codon 146 mutation is a BEAMing dPCR false positive or a both Idylla and NGS false negative. A most sensitive assay as ddPCR would be relevant but the technology was not available in our laboratory. One *KRAS* exon 4, codon 146 mutation was detected with both BEAMing dPCR and NGS and not with ctKRAS cartridge. This sample has been identified to have a low cfDNA concentration, thus using only 1 mL of plasma may not be sufficient for a detection using the Idylla system. Three milliliters of plasma were used for ctDNA extraction for BEAMing dPCR assay and then more DNA was used for amplification and detection with BEAMing, which probably allowed the detection of the mutation for this sample with low allele frequency. Moreover, the main peak size obtained with plasma from patients with a mCRC differ from the one obtained by sonication (Fig 1). Indeed, it has been reported in the literature that ctDNA fragment size depends on tumor type [31,32]. This heterogeneity between cancer types explains the difference between targeted size (based on a mean peak size observed in several cancer type) and observed size (based on our pool of mCRC patients).

Interest for cfDNA application in cancer management has grown these last years. Several studies have demonstrated a high concordance between mutational profiles of candidate genes in matched tumor and plasma DNA samples from mCRC patients. ctDNA should be an easy and reliable tool for targeted therapy introduction [8–10]. *RAS* mutations have been assessed using BEAMing in 146 plasma samples from patients with mCRC in the study published by Grasselli *et al*.; 48% of their samples were found with a mutant allele fraction from 0.01% to 1% [33]. These results show that a sensitive method is required for ctDNA detection for clinical applications. Based on our results, this LOD is reached for *BRAF* mutations with the Idylla system. For *KRAS* and *NRAS* mutations, Idylla system should miss mutations with mutant allele fraction around 0.01% contrary to most sensitive techniques as BEAMing dPCR which reach a sensitivity if 0.01% and digital droplet PCR (ddPCR) which reach a sensitivity of 0.001% [21,34]. Regular amplicon-based NGS assays allow the detection of variants with an allele frequency greater than 2% [35]. Recently, new bioinformatics and library preparation approaches have been described to improve NGS performance for low-allele frequency mutation detection in ctDNA [36–38]. Moreover, NGS methods have highlighted in some cases, non-hotspot mutations with a potential clinical relevance [39–42]. These non-hotspot mutations are not detected by PCR based methods like Idylla because they only focus on hotspot mutations (S4 Table). The main aim of this study was to demonstrate the aptitude of Idylla system to detect hotspot mutations with low allele frequencies. Only few plasma samples from patients with mCRC were available for comparison, which is an actual limit of our study. To assess Idylla system relevance in clinical context, more qualified patient’s samples should be analyzed. Finally, the Idylla system requires a total of 5 minutes hands-on time and 130 minutes to complete an analysis. Assays like droplet-based PCR and NGS require DNA extraction, samples preparation and results interpretation for a total workflow of 1 day for droplet-based PCR, 3 days for BEAMing dPCR and 4 days for NGS.

In conclusion, the Idylla system allows the detection of ctDNA mutations in plasma with limits of detection of 0.1% with a short turnover time. This system can easily be integrated in a routine workflow for plasma analysis and should be considered as well as more sensitive or more explorative assays like ddPCR or NGS respectively. Considering the LOD of the assay and the paucity of ctDNA in some plasma samples, it seems to be still necessary to assess mutational status on tumor tissue when no mutation is detected in plasma.

## Supporting information

S1 Table Detail of culture conditions and quality control testing procedures. (DOCX)

S2 Table All samples dilutions performed for *KRAS* p.(Ala146Thr) and p.(Gly12Val) mutation in *KRAS* gene and tested by ctKRAS mutation assay for samples mimicking plasma. (DOCX)

S3 Table All samples dilutions performed for p.(Val600Glu) mutation in *BRAF* gene and p.(Gln61Lys) in *NRAS* gene and tested by ctNRAS-BRAF mutation assay for samples mimicking plasma. (DOCX)

S4 Table Mutation detection available with Idylla^TM^ ctDNA cartridges. (DOCX)

S5 Table All samples dilutions performed for *KRAS* mutated commercial panel of controls. (DOCX)

S6 Table All samples dilutions performed for *BRAF* and *NRAS* mutated commercial panel of controls. (DOCX)
