# Supplementary material for: Evaluation of KRAS, NRAS and BRAF mutations detection in plasma using an automated system for patients with metastatic colorectal cancer
Source: PLoS One. 2020 Jan 15;15(1):e0227294. doi: 10.1371/journal.pone.0227294 (PMC6961936; doi:10.1371/journal.pone.0227294)
Supplement: S1 Table — (DOCX) [file pone.0227294.s001.docx]

**S1 Table.** Detail of culture conditions and quality control testing procedures.

| **Cell-line** | **Culture medium** | **Incubation** | **CO_2_** | **Quality control testing procedures** |
| --- | --- | --- | --- | --- |
| ML-2 | RPMI^a^ 1640 + 20% FBS^b^ | 37°C | 5% | Mycoplasma testing |
| SW620 | Leibovitz's L-15 + 10% FBS | 37°C | 0% | Mycoplasma testing |
| HT29 | McCoy's 5a Medium Modified + 10% FBS | 37°C | 5% | Mycoplasma testing |

a : RPMI : Roswell Park Memorial Institute

b : FBS : fetal bovine serum
