# Supplementary material for: Evaluation of KRAS, NRAS and BRAF mutations detection in plasma using an automated system for patients with metastatic colorectal cancer
Source: PLoS One. 2020 Jan 15;15(1):e0227294. doi: 10.1371/journal.pone.0227294 (PMC6961936; doi:10.1371/journal.pone.0227294)
Supplement: S2 Table — (DOCX) [file pone.0227294.s002.docx]

**S2 Table.** All samples dilutions performed for *KRAS* p.(Ala146Thr) and p.(Gly12Val) mutation in *KRAS* gene and tested by ctKRAS mutation assay for samples mimicking plasma

|  | **cfDNA concentration^a^**  **ng/mL**  **copies /mL** | **ctDNA**  **concentration^b^**  **ng/mL**  **copies /mL** | **Mutated ctDNA concentration^c^**  **ng/mL**  **copies / mL** | **cfDNA total concentration^d^**  **ng/mL**  **copies /mL** | **Ratio**  **mutated copies / wild-type copies**  **%** | **Cq wild-type (control)^e^** | **Cq mutated^e^** | **Mutation interpretation** |
| --- | --- | --- | --- | --- | --- | --- | --- | --- |
| **p.(Ala146Thr)** | 460.60  138 180 | 0  0 | 0  0 | 460.60  138 180 | 0 | 28.3 | - | Not detected |
|  | 461.06  138 318 | 4.60 x 10^-1^  138 | 2.30 x 10^-1^  69 | 461.52  138 456 | 1/2000  0.050% | 28.7 | 38.6 | Detected |
|  | 460.69  138 208 | 9.00 x 10^-2^  28 | 4.50 x 10^-2^  14 | 460.78  138 236 | 1/10 000  0.010% | 27.7 | 39.2 | Detected |
|  | 460.68  138 203 | 7.70 x 10^-2^  23 | 3.85 x 10^-2^  11.5 | 460.76  138 226 | 1/12 000  0.009% | 29.0 | 39.6 | Detected |
|  | 460.67  138 202 | 7.40 x 10^-2^  22 | 3.70 x 10^-2^  11 | 460.74  138 224 | 1/12 500  0.008% | 29.8 | - | Not detected |
|  | 460.67  138 201 | 7.10 x 10^-2^  21 | 3.55 x 10^-2^  10.5 | 460.74  138 222 | 1/13 000  0.007% | 27.9 | - | Not detected |
|  | 460.67  138 200 | 6.60 x 10^-2^  20 | 3.30 x 10^-2^  10 | 460.736  138 220 | 1/14 000  0.007% | 29.0 | 37.3 | Detected |
|  | 460.67  138 200 | 6.60 x 10^-2^  20 | 3.30 x 10^-2^  10 | 460.736  138 220 | 1/14 000  0.007% | 28.4 | - | Not detected |
|  | 460.66  138 199 | 6.40 x 10^-2^  19 | 3.20 x 10^-2^  9.5 | 460.724  138 218 | 1/14 500  0.007% | 28.2 | - | Not detected |
|  | 460.66  138 198 | 6.10 x 10^-2^  18 | 3.00 x 10^-2^  9 | 460.721  138 216 | 1/15 000  0.006% | 27.2 | - | Not detected |
|  | 460.66  138 197 | 5.80 x 10^-2^  17 | 2.90 x 10^-2^  8.5 | 460.718  138 214 | 1/16 000  0.006% | 28.1 | - | Not detected |
|  | 460.65  138 195 | 5.10 x 10^-2^  15 | 2.50 x 10^-2^  7.5 | 460.702  138 210 | 1/18 000  0.005% | 28.4 | - | Not detected |
|  | 460.65  138 194 | 4.60 x 10^-2^  14 | 2.30 x 10^-2^  7 | 460.696  138 208 | 1/20 000  0.005% | 28.6 |  | Not detected |
| **p.(Gly12Val)** | 460.60  138 180 | 0  0 | 0  0 | 460.6  138 180 | 0 | 28.2 | - | Not detected |
|  | 460.64  138 192 | 4.00 x 10^-2^  12 | 4.00 x 10^-2^  12 | 460.68  138 204 | 1/11 500  0.009% | 28.1 | 38.3 | Detected |
|  | 460.64  138 191 | 3.50 x 10^-2^  10.5 | 3.50 x 10^-2^  10.5 | 460.675  138 202 | 1/13 000  0.008% | 27.9 | 32.8 | Detected |

1. cfDNA concentration: concentration of cfDNA in commercial plasma
2. ctDNA concentration: concentration of ctDNA added in commercial plasma
3. mutated ctDNA concentration = ctDNA concentration for homozygous mutation and ctDNA concentration/2 for heterozygous mutation
4. cf DNA total concentration = cfDNA concentration + ctDNA concentration. cfDNA total number of copies = cfDNA number of copies + ctDNA number of copies
5. Cycle quantification.
