# Supplementary material for: Evaluation of KRAS, NRAS and BRAF mutations detection in plasma using an automated system for patients with metastatic colorectal cancer
Source: PLoS One. 2020 Jan 15;15(1):e0227294. doi: 10.1371/journal.pone.0227294 (PMC6961936; doi:10.1371/journal.pone.0227294)
Supplement: S3 Table — (DOCX) [file pone.0227294.s003.docx]

**S3 Table.** All samples dilutions performed for p.(Val600Glu) mutation in *BRAF* gene and p.(Gln61Lys) in *NRAS* gene and tested by ctNRAS-BRAF mutation assay for samples mimicking plasma

| **Sample** | **cfDNA concentration^a^  ng/mL copies/mL** | **ctDNA**  **concentration^b^**  **ng/mL**  **copies /mL** | **ctDNA concentration^c^ ng/mL copies/mL** | **cfDNA total concentration^d^ ng/mL copies/mL** | **Ratio mutated copies / wild-type copies** | **Cq wild-type^e^ (control)** | **Cq mutated^e^** | **Mutation interpretation** |
| --- | --- | --- | --- | --- | --- | --- | --- | --- |
| **p.Val600Glu** | 460.6 138 180 | 0  0 | 0 | 460.6 138 180 | - | 38.2 | - | Not detected |
|  | 460.67 138 201 | 14.00 x 10^-2^  42 | 7.00 x 10^-2^ 21 | 460.74 138 222 | 1/6 500  0.015% | 37.8 | 53.4 | Detected |
|  | 460.65 138 196 | 10.50 x 10^-2^  32 | 5.25 x 10^-2^ 16 | 460.7025 138 212 | 1/8 750  0.011% | 38.1 | 55.3 | Detected |
|  | 460.65 138 196 | 10.30 x 10^-2^  30 | 5.15 x 10^-2^ 15 | 460.7015 138 211 | 1/9 000  0.011% | 37.7 | - | Not detected |
|  | 460.65 138 195 | 10.00 x 10^-2^  30 | 5.00 x 10^-2^ 15 | 460,70 138 210 | 1/9 250  0.011% | 37.6 | - | Not detected |
|  | 460.65 138 194 | 9.00 x 10^-2^  28 | 4.50 x 10^-2^ 14 | 460.695 138 208 | 1/10 250  0.010% | 37.9 | - | Not detected |
|  | 460.64 138 191 | 7.00 x 10^-2^  21 | 3.50 x 10^-2^ 10.5 | 460.675 138 202 | 1/13 000  0.008% | 38.2 | - | Not detected |
| **p.Gln61Lys** | 460.60 138 180 | 0  0 | 0 0 | 460.60 138 180 | - | 38.2 | - | Not detected |
|  | 460.67 138 201 | 14.00 x 10^-2^  42 | 7.00 x 10^-2^ 21 | 460.74 138 222 | 1/6 500  0.015% | 37.8 | 55 | Detected |
|  | 460.65 138 200 | 13.00 x 10^-2^  40 | 6.50 x 10^-2^ 20 | 460.715 138 220 | 1/7 000  0.014% | 37.7 | - | Not detected |
|  | 460.63 138 199 | 12.66 x 10^-2^  38 | 6.33 x 10^-2^ 19 | 460,6933 138 218 | 1/7 250  0.014% | 37.6 | - | Not detected |
|  | 460.66 138 198 | 12.00 x 10^-2^  36 | 6.00 x 10^-2^ 18 | 460.72 138 216 | 1/7 500  0.013% | 37.9 | - | Not detected |
|  | 460.65 138 196 | 10.50 x 10^-2^  32 | 5.25 x 10^-2^ 16 | 460.7025 138 212 | 1/8 750  0.011% | 38.1 | - | Not detected |
|  | 460.62 138 180 | 3.34 x 10^-2^  10 | 1.67 x 10^-2^ 5 | 460.6367 138 185 | 1/27 500  0.004% | 38.2 | - | Not detected |

1. cfDNA concentration: concentration of cfDNA in commercial plasma
2. ctDNA concentration: concentration of ctDNA added in commercial plasma
3. mutated ctDNA concentration = ctDNA concentration for homozygous mutation and ctDNA concentration/2 for heterozygous mutation
4. cf DNA total concentration = cfDNA concentration + ctDNA concentration. cfDNA total number of copies = cfDNA number of copies + ctDNA number of copies
5. Cycle quantification
