# Supplementary material for: Evaluation of KRAS, NRAS and BRAF mutations detection in plasma using an automated system for patients with metastatic colorectal cancer
Source: PLoS One. 2020 Jan 15;15(1):e0227294. doi: 10.1371/journal.pone.0227294 (PMC6961936; doi:10.1371/journal.pone.0227294)
Supplement: S4 Table — (DOCX) [file pone.0227294.s004.docx]

**S4 Table.** Mutation detection available with Idylla^TM^ ctDNA cartridges

| **ctKRAS mutations detection** | | | |
| --- | --- | --- | --- |
| Codon 12 (exon 2) | p.(Gly12Cys) | (c.34G>T) | |
|  | p.(Gly12Arg) | (c.34G>C) | |
|  | p.(Gly12Ser) | (c.34G>A) | |
|  | p.(Gly12Ala) | (c.35G>C) | |
|  | p.(Gly12Asp) | (c.35G>A) | |
|  | p.(Gly12Val) | (c.35G>T) | |
| Codon 13 (exon 2) | p.(Gly13Asp) | (c.38G>A) | |
| Codon 59 (exon 3) | p.(Ala59Thr) | (c.176C>A) | |
|  | p.(Ala59Glu) | (c.176C>G) | |
|  | p.(Ala59Gly) | (c.175G>A) | |
| Codon 61 (exon 3) | p.(Gln61Lys) | (c.181C>A; c.180_181delinsAA) | |
|  | p.(Gln61Leu) | (c.182A>G) | |
|  | p.(Gln61Arg) | (c.182A>T) | |
|  | p.(Gln61His) | (c.183A>C; c.183A>T) | |
| Codon 117 (exon 4) | p.(Lys117Asn) | (c.351A>C; c.351A>T) | |
| Codon 146 (exon 4) | p.(Ala146Pro) | (c.436G>C) | |
|  | p.(Ala146Thr) | (c.436G>A) | |
|  | p.(Ala146Val) | (c.437C>T) | |
| **ctBRAF mutations detection** | |  | |
| Codon 600 | p.(Val600Glu) | (c.1799T>A; c.1799_1800delinsAA) | |
|  | p.(Val600Asp) | (c.1799_1800delinsAC) | |
|  | p.(Val600Lys) | (c.1798_1799delinsAA) | |
|  | p.(Val600Arg) | (c.1798_1799delinsAG) | |
| **ctNRAS mutations detection** | | | |
| Codon 12 (exon 2) | p.(Gly12Cys) | | (c.34G>T) |
|  | p.(Gly12Ser) | | (c.34G>A) |
|  | p.(Gly12Asp) | | (c.35G>A) |
|  | p.(Gly12Ala) | | (c.35G>C) |
|  | p.(Gly12Val) | | (c.35G>T) |
| Codon 13 (exon 2) | p.(Gly13Asp) | | (c.38G>A) |
|  | p.(Gly13Val) | | (c.38G>T) |
|  | p.(Gly13Arg) | | (c.37G>C) |
| Codon 59 (exon 3) | p.(Ala59Thr) | | (c.175G>A) |
| Codon 61 (exon 3) | p.(Gln61Lys) | | (c.181C>A) |
|  | p.(Gln61Leu) | | (c.182A>T) |
|  | p.(Gln61Arg) | | (c.182A>G) |
|  | p.(Gln61His) | | (c.183A>C; c.183A>T) |
| Codon 117 (exon 4) | p.(Lys117Asn) | | (c.351G>C; c.351G>T) |
| Codon 146 (exon 4) | p.(Ala146Thr) | | (c.436G>A) |
|  | p.(Ala146Val) | | (c.437C>T) |
