# Supplementary material for: Evaluation of KRAS, NRAS and BRAF mutations detection in plasma using an automated system for patients with metastatic colorectal cancer
Source: PLoS One. 2020 Jan 15;15(1):e0227294. doi: 10.1371/journal.pone.0227294 (PMC6961936; doi:10.1371/journal.pone.0227294)
Supplement: S5 Table — (DOCX) [file pone.0227294.s005.docx]

**S5 Table.** All samples dilutions performed for *KRAS* mutated commercial panel of controls

|  | **Volume of control  (µL) in 1 mL of commercial plasma** | **Number of Mutated copies in the sample** | **Ratio mutated copies /  wild-type copies %** | **Cq wild-type^a^ (control)** | **Cq mutated^a^** | **Mutation interpretation** |
| --- | --- | --- | --- | --- | --- | --- |
|  |  |  |  |  |  |  |
|  |  |  |  |  |  |  |
|  |  |  |  |  |  |  |
|  |  |  |  |  |  |  |
| p.(Gly12Asp) | 60.60 | 138 | 0.1% | 24.7 | 30.61 | Detected |
|  | 30.30 | 69 | 0.05% | 25.5 | 33.36 | Detected |
|  | 12.12 | 28 | 0.02% | 26.3 | - | Not detected |
|  | 6.06 | 14 | 0.01% | 27.4 | - | Not detected |
| p.(Gly12Ser) | 60.60 | 138 | 0.1% | 25.3 | 32.6 | Detected |
|  | 30.30 | 69 | 0.05% | 26.0 | 34.1 | Detected |
|  | 15.15 | 35 | 0.025% | 26.5 | - | Not detected |
| p.(Gly12Val) | 60.60 | 138 | 0.1% | 25.3 | 33.4 | Detected |
|  | 30.30 | 69 | 0.05% | 26.0 | 36.7 | Detected |
|  | 15.15 | 35 | 0.025% | 26.4 | 36.5 | Detected |
|  | 9.09 | 21 | 0.015% | 27.0 | 36.97 | Detected |
|  | 6.06 | 14 | 0.01% | 27.2 | - | Not detected |
| p.(Gly13Asp) | 90.90  60.60  45.45 | 197  138  104 | 0.15%  0.1%  0.075% | 25.2  24.9  25.6 | 30.3  30.8  - | Detected  Detected  Not detected |
|  | 30.30 | 69 | 0.05% | 25.7 | - | Not detected |
|  | 12.12 | 28 | 0.02% | 26.7 | - | Not detected |
|  | 9.09 | 21 | 0.015% | 26.9 | - | Not detected |
