# Supplementary material for: Evaluation of KRAS, NRAS and BRAF mutations detection in plasma using an automated system for patients with metastatic colorectal cancer
Source: PLoS One. 2020 Jan 15;15(1):e0227294. doi: 10.1371/journal.pone.0227294 (PMC6961936; doi:10.1371/journal.pone.0227294)
Supplement: S6 Table — (DOCX) [file pone.0227294.s006.docx]

**S6 Table.** All samples dilutions performed for *BRAF* and *NRAS* mutated commercial panel of controls

|  | **Volume of control  (µL) in 1 mL of commercial plasma** | **Number of Mutated copies in the sample** | **Ratio mutated copies /  wild-type copies %** | **Cq wild-type^a^ (control)** | **Cq mutated^a^** | **Mutation interpretation** |
| --- | --- | --- | --- | --- | --- | --- |
|  |  |  |  |  |  |  |
|  |  |  |  |  |  |  |
|  |  |  |  |  |  |  |
|  |  |  |  |  |  |  |
| p.(Val600Glu) | 6.06  4.54 | 14  10 | 0.01%  0.0075% | 37./  37.1 | 55.1  52.7 | Detected  Detected |
|  | 3.03 | 7 | 0.005% | 37.3 | - | Not detected |
| p.(Gly12Asp) | 151.50  121.20 | 690  552 | 0.5%  0.4% | 34.6  35.0 | 47.0  50.6 | Detected  Detected |
|  | 90.90 | 414 | 0.3% | 35.1 | - | Not detected |
|  | 60.60 | 276 | 0.2% | 35.9 | - | Not detected |
|  | 30.30 | 138 | 0.1% | 36.4 | - | Not detected |
| p.(Gly12Val) | 151.50 | 690 | 0.5% | 35.0 | 43.8 | Detected |
|  | 90.90 | 414 | 0.3% | 35.2 | 45.1 | Detected |
|  | 30.30 | 138 | 0.1% | 36.5 | 50.7 | Detected |
|  | 15.15 | 69 | 0.05% | 36.7 | - | Not detected |
| p.(Gln61Arg) | 90.90 | 414 | 0.3% | 35.3 | 44.3 | Detected |
|  | 30.30 | 138 | 0.1% | 37.6 | 46.4 | Detected |
|  | 15.15 | 69 | 0.05% | 37.4 | - | Not detected |
| p.(Gln61Lys) | 121.2  90.90  75.75 | 552  414  345 | 0.4%  0.3%  0.25% | 35.1  35.8  35.5 | 48.42  - | Detected  Detected  Not detected |
|  | 60.60 | 276 | 0.2% | 35.4 | - | Not detected |
|  | 30.30 | 138 | 0.1% | 36.9 | - | Not detected |
